# Torque generating properties of *Tetrahymena* ciliary three-headed outer-arm dynein

**DOI:** 10.1038/s41598-022-21001-0

**Published:** 2022-10-06

**Authors:** Shin Yamaguchi, Masahiko Yamagishi, Junichiro Yajima

**Affiliations:** 1 Department of Life Sciences, Graduate School of Arts and Sciences, The University of Tokyo, 3-8-1 Komaba, Meguro-ku, Tokyo, 153-8902 Japan; 2 Komaba Institute for Science, The University of Tokyo, 3-8-1 Komaba, Meguro-ku, Tokyo, 153-8902 Japan; 3 Research Center for Complex Systems Biology, The University of Tokyo, 3-8-1 Komaba, Meguro-ku, Tokyo, 153-8902 Japan; 4 Universal Biological Institute, The University of Tokyo, Bunkyo-ku, Tokyo, 113-0033 Japan

**Keywords:** Motor protein function, Dynein

## Abstract

Eukaryotic cilia/flagella are cellular bio-machines that drive the movement of microorganisms. Molecular motor axonemal dyneins in the axoneme, which consist of an 9 + 2 arrangement of microtubules, play an essential role in ciliary beating. Some axonemal dyneins have been shown to generate torque coupled with the longitudinal motility of microtubules across an array of dyneins fixed to the coverglass surface, resulting in a corkscrew-like translocation of microtubules. In this study, we performed three-dimensional tracking of a microbead coated with axonemal outer-arm dyneins on a freely suspended microtubule. We found that microbeads coated with multiple outer-arm dyneins exhibited continuous right-handed helical trajectories around the microtubule. This unidirectional helical motion differs from that of other types of cytoplasmic dyneins, which exhibit bidirectional helical motility. We also found that, in an in vitro microtubule gliding assay, gliding microtubules driven by outer-arm dyneins tend to turn to the left, causing a curved path, suggesting that the outer-arm dynein itself is able to rotate on its own axis. Two types of torque generated by the axonemal dyneins, corresponding to the forces used to rotate the microtubule unidirectionally with respect to the long and short axes, may regulate ciliary beating with complex waveforms.

## Introduction

The success of the autonomous beating of eukaryotic cilia/flagella depends on the coordinated activity of microtubule-based molecular motors, axonemal dyneins, which use ATP as energy for generating directed forces^1^. Axonemal dyneins consist of one outer-arm dynein (OAD) and several inner-arm dyneins (IADs), according to their location within the axoneme^2^. OAD is an essential axonemal dynein involved in the beating motion of cilia and flagella^3^. OAD forms either heterotrimers or heterodimers consisting of single sets of a multi-subunit protein complex containing three (in protists)^4^ or two (in metazoans)^5^ distinct motor domains of dynein heavy chains depending on the species, and are positioned at the A-tubule of the nine-peripheral microtubule doublets of the axoneme every 24 nm^6–8^. Each motor domain, which contains functional regions, such as the ATPases associated with the diverse cellular activities (AAA^+^) ring and microtubule-binding domain (MTBD) for microtubule binding ability^9^, exhibits repetitive cycles of ATP-dependent interaction with the B-tubule of the nine-peripheral microtubules to generate sliding forces that can drive the propagated bending of cilia and flagella^10^.

The motor activity of *Tetrahymena thermophila* OAD (also known as 22S dynein) is relatively well-studied. OAD is isolated from the outer rows of arms of ciliary axonemes and contains three distinct heavy chains (α, β, γ) forming a “flower bouquet structure” shown by electron microscopy, where their tail domains of three heavy chains are tied up and the motor domains are spread apart^4,11,12^. Recently, high-resolution structures of *T. thermophila* OAD arrays bound to doublet microtubules showed that the three motor domains of OAD are stacked roughly perpendicular to the longitudinal axis of the microtubules^13,14^. The minus-end-directed microtubule motor activity of isolated OAD molecules has been demonstrated using in vitro microtubule gliding assays in which OAD molecules are fixed to a glass surface^15,16^. Single molecules of OAD attached to a microbead have been shown to move processively along a microtubule only at low ATP concentrations with a maximum force of ~ 5 pN using an optical trapping microscope^17^.

As a mechanical property of dyneins, both OAD (*Tetrahymena*)^18^ and some IADs (*Tetrahymena* and *Chlamydomonas*)^15,19^ have been reported to generate torque, which, together with the microtubule longitudinal motility across an array of motors fixed to a surface in an in vitro microtubule motility assay, results in a corkscrew-like motion of microtubule gliding. Both OAD and IADs showed right-handed corkscrewing pitch within a range of 0.2–0.8 μm. Cytoplasmic dynein (CD), which belongs to a different class from axonemal dyneins, is responsible for intracellular transport processes, such as cargo/organelle transport^20^, and also drove gliding microtubules to rotate unidirectionally in an in vitro microtubule gliding assay^21^. In contrast, a microbead coated with CD molecules showed bidirectional helical movement around the microtubule longitudinal axis, suggesting the flexibility of CD to transport cargo in dense cellular environments^22^. However, whether bidirectional helical motion is common to both axonemal and cytoplasmic dyneins remains unknown because the three-dimensional (3D) trajectories of axonemal dyneins have not been examined.

In this study, we tracked the 3D motion of microbeads coated with *T. thermophila* OAD (22S dynein) molecules around a suspended microtubule to examine helical motion. We found that OAD molecule-coated microbeads moved helically in a right-handed manner around the suspended microtubule, which differs from the bidirectional helical motion of CD molecule- coated microbeads^22^. Moreover, in an in vitro classical microtubule gliding assay, where OAD molecules were fixed to the glass coverslip surface, paths of gliding microtubules were circular as observed for *Chlamydomonas* IAD-d or -g driven microtubule gliding motility^23^. The unique motile properties of axonemal dyneins may be involved in a specific function of the dyneins for cilia and flagella beating.

## Results

### Quantification of helical motion of microbeads coated with OAD molecules around a microtubule surface using 3D tracking

**Figure 1:**
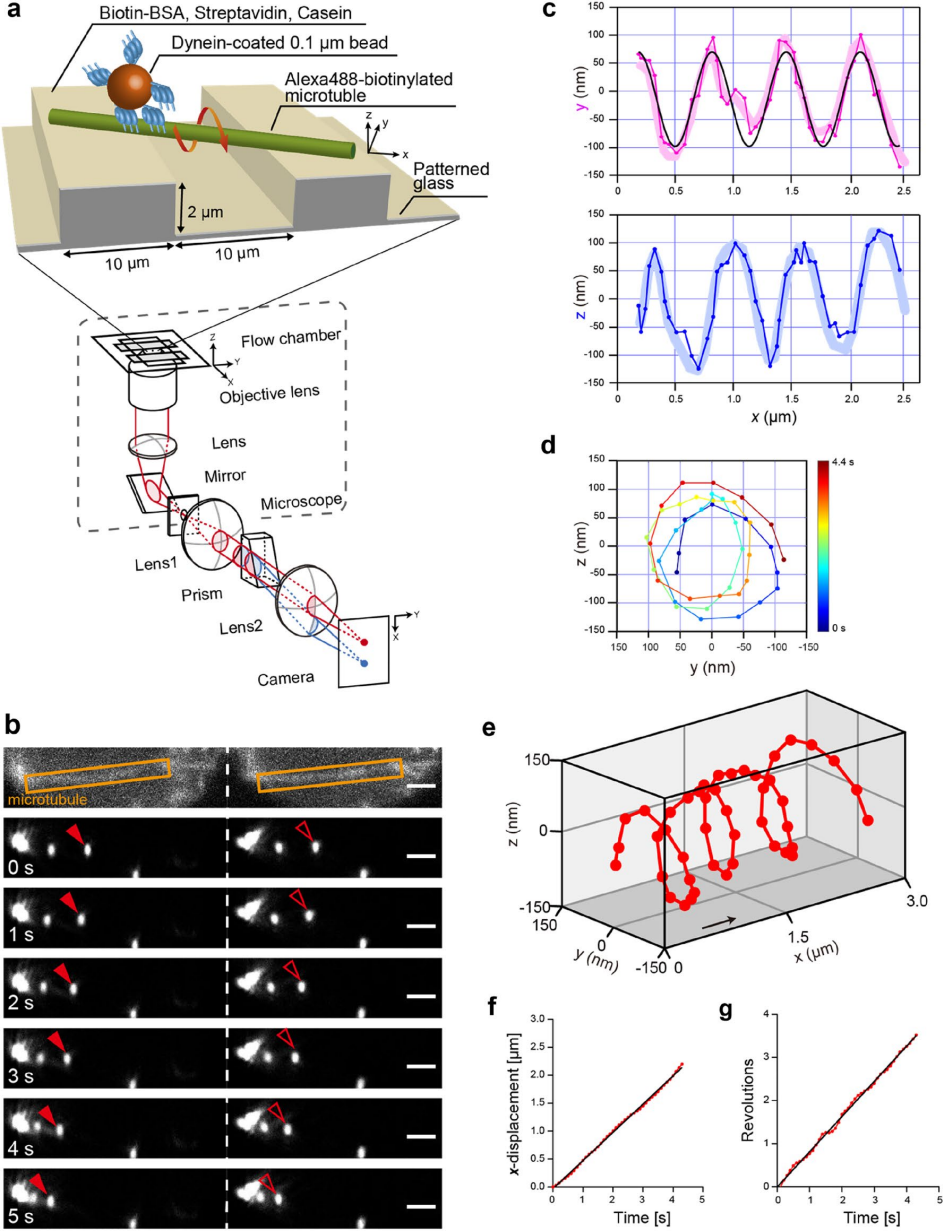
A 3D movement of *Tetrahymena thermophila* OAD 22S dynein around the surface of suspended microtubules. (**a**) Schematic of the experimental setup using a 3D prismatic optical tracking (*tPOT*) microscope. The glass substrate was patterned forming 2-µm-high, 10-µm-wide parallel walls with a 10-µm gap. Microtubules were suspended between the walls using biotin-avidin linkage. The multiple OAD molecule-coated microbeads were allowed to move freely over the microtubule surface. (**b**) Sequential pair of split images of the microbeads obtained using the *tPOT* microscope. The top image displays an Alexa488-labeled microtubule, and the others display an OAD-coated microbead translocated along the suspended microtubule. The solid and open red arrowheads indicate a pair of images of a microbead split by the prism. Scale bar, 2 μm. (**c**) (Top) *x*–*y* (pink) and (bottom) *x*–*z* (blue) trajectories of the microbead driven by OAD molecules shown in (**b**). Solid lines with dots show the data acquired at 10 frames s^-1^, whereas the thick lines show the data averaged over three frames. The helical pitch was obtained by fitting the *x*–*y* trace of the microbead with a sine function (black), giving a value of 0.62 µm. (**d**) *y–z* trajectory of the microbead shown in (**b**). The *y–z* trajectory displays a clockwise rotation when looking in the direction of the forward translocation. Color indicates the observation time (see color bar). (**e**) 3D plot of the microbead shown in (**b**) reveals a right-handed helical motion along the microtubule. The black arrow indicates the approximate displacement over 1 s. (**f**) Time course of *x*-displacement of the microbead shown in (**b**). The longitudinal velocity was obtained from the slope by fitting the *x-*displacement versus time of the microbead (red) with a line function (black line), giving a value of 0.50 µm s^-1^. (**g**) Time course of revolutions of the microbead shown in (**b**). The rotational velocity was obtained from the slope by fitting the revolutions versus time of the microbead (red) with a line function (black line), giving a value of 0.82 rev s^-1^.

To gain insights into the torque generated during the minus-end-directed movement of axonemal dynein, the 3D motion of a fluorescent microbead (0.1 µm in diameter) driven by multiple OAD molecules was examined. To this end, three-headed OAD (22S dynein) molecules were extracted from *T. thermophila* ciliary axonemes and attached to microbeads. In an experimental geometry where microtubules are suspended away from the glass coverslip surface, microbeads bound to multiple dynein motors were allowed to move freely over the microtubule surface (Fig. 1a). 3D movements of the microbeads around the microtubule surface were tracked using a three-dimensional prismatic optical tracking (*tPOT*) microscope (Fig. 1a), originally developed by us to track single fluorescent molecules^24^, quantum dots^25^, microbeads^26^, or cells^27^. Our *tPOT* achieved nanometer-order accuracy in 3D positioning of a microbead fixed on the suspended microtubule (see supplementary Fig. 1 in Maruyama et al.^28^). To quantify the motion of OAD molecules along the microtubule, the *xyz* positions of the OAD-coated microbeads were determined. The *x* and *y* positions of the microbeads were calculated from the average of the two positions of the microbeads in each image split by prism, and the *z* position of the microbeads was obtained from the difference between the two *y*-positions of the microbeads (Fig. 1b and Supplementary Movie 1). Both *x–y* (Fig. 1c pink) and *x–z* trajectories (Fig. 1c blue) of a microbead coated with OAD molecules at low ATP concentration (10 μM ATP) displayed oscillations. These trajectories with a phase lag between the sinusoidal oscillations indicated that the microbeads moved helically around the longitudinal axis of the microtubule. The corresponding *y–z* trajectory was circular (Fig. 1d), and the handedness of the helical motion was right-handed. Helical handedness was also confirmed by a 3D plot (Fig. 1e). For the microbead in Fig. 1b, the helical pitch, longitudinal, and rotational velocities of the helical motion were 0.62 μm, 0.50 μm s^−1^ and 0.82 rev s^−1^, respectively (Fig. 1c, f, g).

### OADs display unidirectional right-handed helical motions around a suspended microtubule

**Figure 2:**
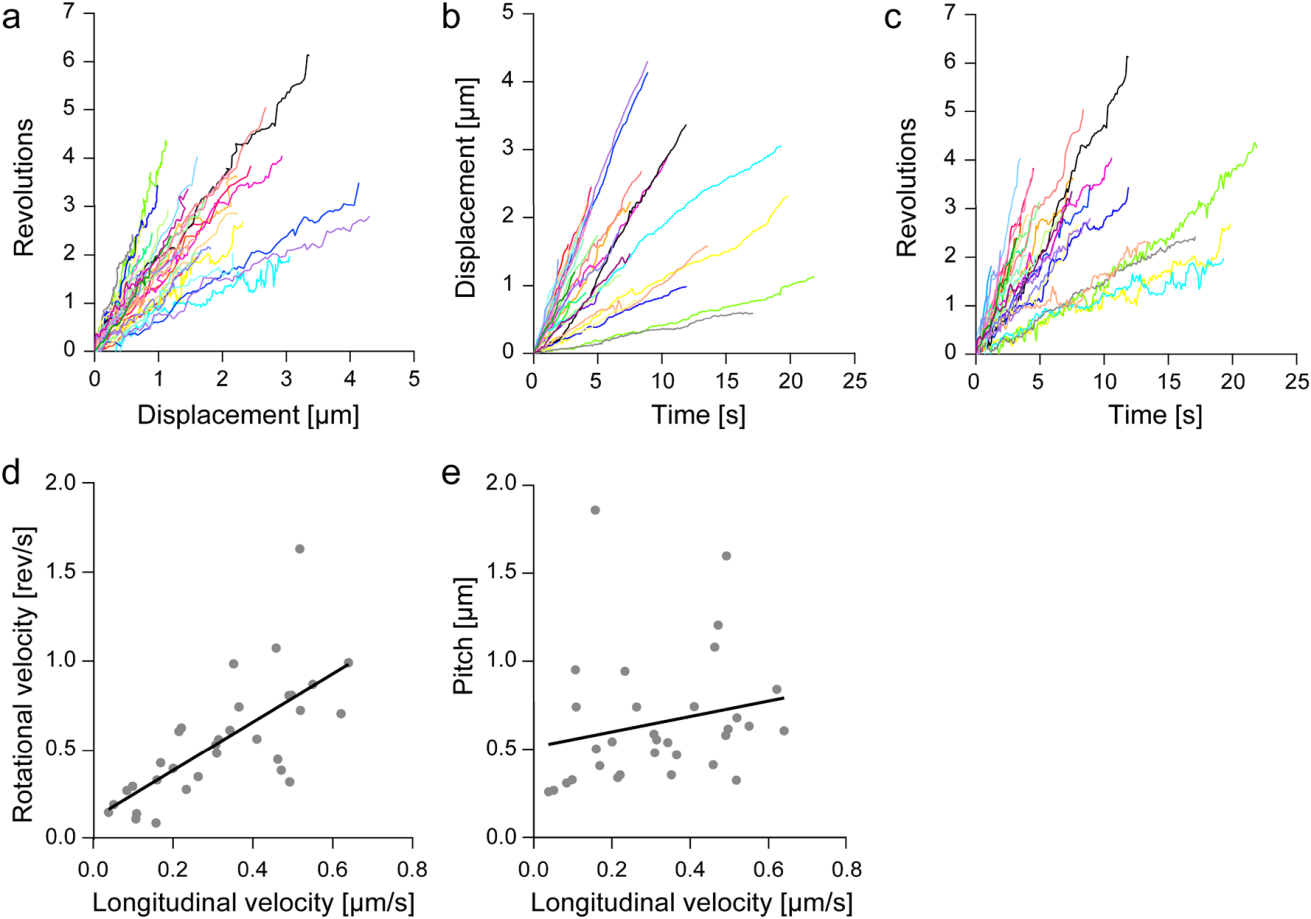
Helical motion of OAD-coated microbeads. (**a**) Cumulative revolutions plotted versus longitudinal displacement for OAD-coated microbeads (*n* = 32). The rotation to the right with respect to the movement in the longitudinal direction is expressed as a positive number. The average pitch was + 0.65 ± 0.36 µm (mean ± SD). (**b, c**) Time course of longitudinal distances (**b**) and revolutions (**c**) of the microbeads (*n* = 32). Individual trajectories in (**b**) and (**c**) were fitted with line functions to determine the longitudinal and rotational velocities, respectively. Average longitudinal and rotational velocities were 0.32 ± 0.17 µm s^−1^ and 0.55 ± 0.33 rev s^−1^, respectively. (**d, e**) Correlation of rotational velocity and helical pitch with longitudinal velocity. The correlation coefficients, *R* and linear fit, *R*^*2*^, of the longitudinal and rotational velocities, and longitudinal velocity and helical pitch were *R* = 0.71; *R*^*2*^ = 0.51 (**d**) and *R* = 0.21; *R*^*2*^ = 0.04 (**e**) (*n* = 32), respectively.

Based on the plot of revolution versus *x*-displacement, the trajectories of the microbeads coated with OAD molecules were always right-handed helical paths (*n* = 32 microbeads; Fig. 2a). The handedness of the helical motions of microbeads driven by OAD molecules was the same as that of the corkscrewing microtubule driven by OAD^18^, IAD 14S dyneins^15^, IAD dynein-a^29^, and CD^21^ in microtubule corkscrewing assays. The handedness of helical motions of microbeads driven by OAD molecules was unidirectional for each microbead, which differs from the bidirectional helical motions of microbeads driven by CD molecules around the microtubule longitudinal axis^22^. However, the trajectories were found to have a wide zone (helical pitch; + 0.3 to + 1.9 μm, the plus sign refers to a right-handed helical pitch), as observed in the trajectories of CD molecules. The mean helical pitch of OAD-coated microbeads was estimated to be + 0.65 ± 0.36 μm (mean ± standard deviation, SD, *n* = 32). From the time course of longitudinal displacement and rotation along a microtubule for individual microbeads, the longitudinal and rotational velocities for microbeads that made >  ~ 2 revolutions were determined (Fig. 2b, c). The distribution in longitudinal and rotational velocity varied between 0.04–0.64 μm s^−1^, and between 0.09–1.62 rev s^−1^, with a mean longitudinal velocity of 0.32 ± 0.17 μm s^−1^ (mean ± SD, *n* = 32) and a mean rotational velocity of 0.55 ± 0.33 revolutions s^−1^ (mean ± SD, *n* = 32), respectively. An increase in the longitudinal velocity, with a subsequent increase in the rotational velocity, was observed (Fig. 2d). However, there was only a small effect on helical pitch (Fig. 2e). This result indicates that both longitudinal and rotational velocities were increased to the same extent, with the ratio of longitudinal to rotational velocity remaining constant.

### Observation of circular motion of gliding microtubules driven by OAD

**Figure 3:**
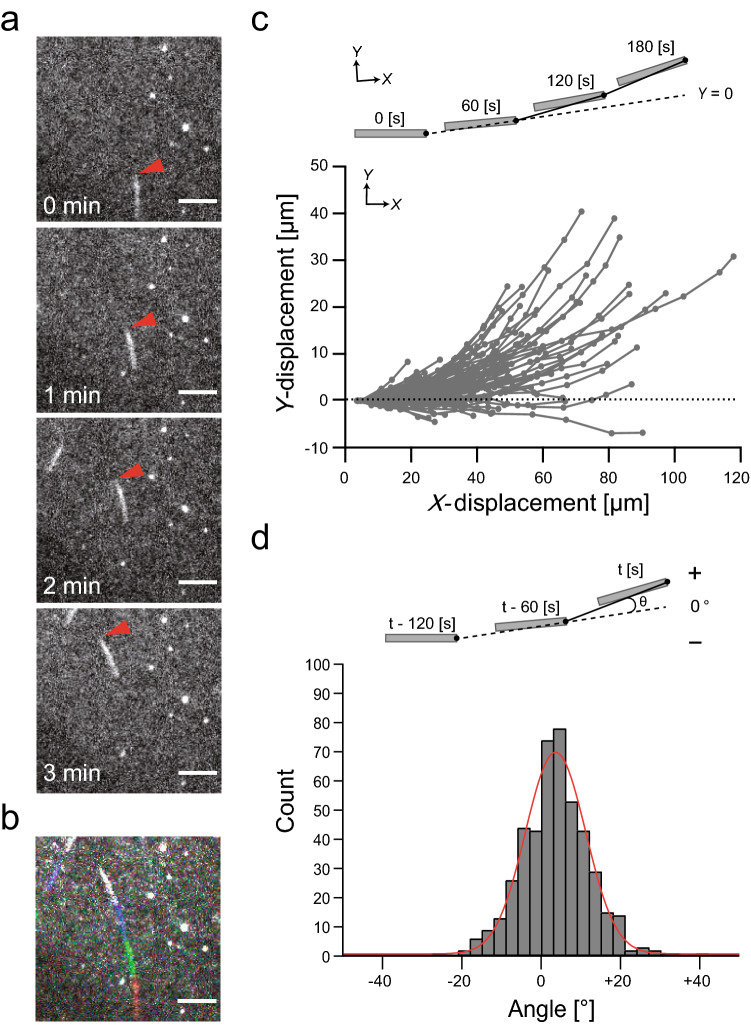
Circumferential gliding movement of microtubules driven by OAD. (**a**) Sequential images of rhodamine-labeled microtubule gliding driven by OAD. The solid arrowheads indicate the leading tip of the microtubule. Scale bar, 10 μm. (**b**) Superimposing successive images of a gliding microtubule driven by OAD taken every 1 min for 3 min. The first image is shown in red. Scale bar, 10 μm. (**c**) (Top) For each microtubule, the line connecting the microtubule tip in the first and second frames was defined as the *y*-axis (dotted line, *y* = 0), and the *x–y* position of the microtubule tip in each frame measured every 60 s was plotted. (Bottom) *x*–*y* trajectories of the tip of the gliding microtubules driven by OAD. The *x–y* position of the forward tip of the gliding microtubule to the left with respect to the gliding movement in the forward direction was expressed as a positive number (*n* = 97 microtubules). (**d**) (Top) The angle between the line (solid line) defined by the front positions of the microtubule in each frame and in one previous frame, and the line (dotted line) defined by the front positions of the microtubule in one previous frame and in two previous frames were measured. (Bottom) Histogram of the direction angle of gliding microtubules every 60 s. The mean angular change was obtained from Gaussian fitting to the histogram of angular change per frame, giving values of + 3.4° every 60 s (*n* = 458 angles). The plus (+) and minus (−) signs refer to the left and right side of the angles.

In an in vitro microtubule corkscrewing assay, OAD has been shown to drive the right-handed corkscrewing motion of microtubules with a corkscrewing pitch of + 0.76 μm^18^, which is consistent with the right-handed unidirectional helical pitch of microbeads coated with OAD (Fig. 2a). Such observations of unidirectional corkscrewing motions and helical motions driven by OAD molecules imply that the stepping of the motor head of a three-headed dynein tends, on average, to bind a right-forward tubulin of the microtubule, producing a unidirectional lateral force perpendicular to the longitudinal axis of the microtubule. Such a unidirectional transverse force might allow gliding of the microtubules to drive the lateral transition. Given that a microtubule (circumference of 0.08 μm) rotates once for every 0.76 μm it advances, it is estimated that a sideward displacement is ~ 1 μm for every ~ 10 μm it advances^15^. To examine whether microtubules translocate sideways during forward motion, we observed the movement of individual microtubules over a distance of 20–120 μm in the presence of 10 μM ATP in a classical microtubule gliding assay (Fig. 3a and Supplementary Movie 2). The concentration of loaded microtubules was sufficiently low to prevent the microtubules from colliding with each other. We found that most microtubules moved forward smoothly and did not show lateral movement. This is consistent with previous reports using *T. thermophila* IAD (14S dynein mixture)^15^, *Chlamydomonas* IAD-d, or -g^23^, which also drove microtubule corkscrewing^15,19^. However, tracking the forward position of the microtubules revealed that gliding microtubules tended to orbit to the left (Fig. 3b, c). The paths of the microtubules every 60 s were also analyzed by measuring the angle at which the forward tip position of the microtubule in each frame changed relative to the forward tip position of the microtubule in the previous frame (Fig. 3d top). The mean angular change of the forward tip position of the microtubules every 60 s was obtained from Gaussian fitting to the histogram of the angular change, giving values of 3.4° every 60 s (Fig. 3d bottom). This corresponds to a radius of curvature of ~ 235 μm, which is consistent with the value of a previous report on *Chlamydomonas* IAD-g^23^. Thus, OAD caused microtubules to turn to the left in our setup. Consistent with a previous report using IADs^23^, this indicates that the same extent of microtubule circular motion is caused by an intrinsic property of the motor activity of both IAD and OAD.

## Discussion

Dyneins are classified into two classes, axonemal and cytoplasmic dyneins, which are structurally and functionally distinct. Axonemal dyneins drive the high-speed oscillatory beating of motile cilia/flagella, while cytoplasmic dyneins are responsible for various intracellular transport processes, such as the mitotic spindle function and trafficking of various organelles. Thus, elucidating the characteristic activities of these two types of dynein motors is important for understanding a variety of cellular processes. As one mode of motility, in the microtubule corkscrewing assay, in which microtubules move across an array of motors fixed to the glass surface, both axonemal and cytoplasmic dyneins generate torque, which, together with the microtubule gliding motility, causes right-handed corkscrewing microtubule motions^15,18,19,21^. In contrast, when dynein-coated microbeads were allowed to move over the whole surface of the microtubule suspended away from the cover glass surface, the microbeads showed helical motion along the microtubules. In such a microbead assay, OAD-driven microbeads exhibited unidirectional, right-handed helical motions (Fig. 1), whereas CD-driven microbeads exhibited bidirectional helical motions^22^. Although the assay geometries and the particular forces acting either in the microtubule corkscrewing assay or in the microbead assay differ, the most likely factor affecting motility in these two in vitro systems is the difference in the number of dynein molecules involved in the movement. In a microtubule corkscrewing assay, hundreds to thousands of dynein molecules are involved in the corkscrewing motion of a microtubule, whilst in the microbead assay, a small number of dynein molecules act on the helical movement of a microbead. In such a microbead assay using CD, fluctuations due to the small number of dynein molecules involved in movement are likely to be apparent in the left and right directions as the CD molecule moves forward, resulting in bidirectional helical motions. The ability of switching directions in their sideways movement in a highly crowded environment in cells might facilitate the transport of CD-driven cargo, as discussed previously^22,30^. Bidirectional off-axis motions of motor-coated microbeads have also been reported for other microtubule-based motors, such as kinesin-6 MKLP1-CYK4 complex^28^ and kinesin-8 Kip1^31^, which work in mitotic spindles. In contrast, even with a small number of molecules where fluctuations in the step direction are likely to appear, microbeads coated with multiple OAD molecules displayed unidirectional right-handed helical motions along a suspended microtubule (Fig. 2a). Since the tail domains of OAD molecules are fixed to the A-tubule of nine peripheral microtubule doublets and their stalk head domains interact with the B-tubule in the axoneme, it may efficiently fill the sliding of doublet microtubules by stroking in a specific direction, which is suitable for functioning in a beating organelle. This will be clarified by analyzing the direction in which single molecules of OAD move by single-molecule assays, as has been done for CD^32^.

Recently, a structural study of purified *Tetrahymena* OAD bound to doublet microtubules demonstrated that free OADs had the tendency to bind the doublet microtubule in an array via tail-to-head interactions^14^. In our experiments, since a part of the OAD domains (probably a part of the tail domain) was non-specifically anchored to the microbead in a random position, it is unlikely that OAD arrays were formed on the microtubules. Moreover, the structural study demonstrated that each motor head of a three-headed OAD tended to bind to doublet microtubule protofilaments with specific inter-protofilament angles^14^. However, in our experiments, singlet microtubules were self-assembled in vitro from purified porcine brain tubulin dimers, so that angle differences between protofilaments would not form as in doublet microtubules. Around the lattice surface of freely suspended, singlet microtubules, OAD-coated microbeads could move to the neighboring protofilament on the right to achieve right-handed helical motions along the suspended microtubule (Figs. 1 and 2). Unlike the behavior of OADs on singlet microtubules, on doublet microtubules in axonemes, each OAD motor head may track the same protofilament. In this case, the effect of the torsional strokes of individual heads of OAD whose tail region is anchored to the A-tubule can efficiently twist the microtubules to control complex ciliary beats. Further studies with three-dimensional and higher temporal observations of the in vivo dynamics of OAD and doublet microtubules are required to reveal the biological roles of torque generation driven by OAD.

Using an in vitro microtubule gliding assay for long-term observation of microtubule movement in a relatively wide field of view, we have shown that OAD has the ability to render gliding microtubule orbits counterclockwise in our setup, while OAD does not cause microtubules to translocate sideways (i.e., rolling) during forward motion (Fig. 3). Given that a similar microtubule circumferential orbital motion has been reported only in *Chlamydomonas* IADs^23^, which also drive right-handed corkscrewing microtubule motions^19^, this motility mode may be unique to specific axonemal dyneins (i.e., both IAD and OAD) that also have the ability to corkscrew microtubules. As the mechanism of OAD-driven microtubule corkscrewing motion without lateral (rolling) motion remains unclear, the mechanism by which OAD causes circular motion in gliding microtubules remains unclear as well. The possible explanations are as follows: the direction of the lateral force acting when OAD molecules drive the right-handed corkscrewing motion of the microtubule and the direction of the lateral force acting when OAD molecules drive the microtubule gliding in the direction are identical, so that the lateral force exerted only by OAD molecules interacting with the tubulins near the leading plus-end of the microtubule, which has high freedom of movement, causes the tip of the microtubule to bend slightly to the left; therefore, the gliding microtubule shows counterclockwise arc-shaped trajectories. Another possibility is that, in addition to the forces acting along or around the longitudinal axis of the microtubule, which cause a unidirectional corkscrewing motion of microtubules or helical motion of dynein-coated microbeads along microtubules (Figs. 1 and 2), OAD may have an inherent yawing force (another type of torque); in other words, OAD itself has the ability to rotate along its own axis, which can bend a microtubule at its point of attachment to OAD. Such torque may cause OAD molecules to drive the gliding of microtubules in unidirectional circular paths (Fig. 3). A similar possibility was discussed in a study by Kikushima and Kamiya using *Chlamydomonas* IADs^23^, although the direction of the yawing force was the opposite. The difference may be due simply to the different yaw characteristics or number of motor domains in one-headed IADs and three-headed OADs, or potentially due to different glass surface conditions. How this motion is achieved is unclear and requires further investigation, such as polarization measurements that can detect changes in the rotational direction in a single OAD molecule, and force measurement of yawing generated by a small number of OAD molecules.

In summary, in contrast to CD, which produces bidirectional helical motions around the microtubule surface, we found that *T. thermophila* axonemal OAD molecule-coated microbeads exhibited unidirectional, right-handed helical motions. Despite the similarity of the 3D structures of motor domains of the two types of dyneins (axonemal and cytoplasmic dynein), their helical motion behavior differs, suggesting particular roles for OAD in driving axonemal beating. In contrast to previous reports^15^, we also found that *T. thermophila* OAD molecules were able to drive the counterclockwise, circular motility of the microtubules, which is similar to that previously reported for *Chlamydomonas* IAD. Further studies on torque generation by both OAD and IAD in the axoneme during beating in three dimensions are required to reveal the particular role of axonemal dynein that regulates cilia/flagella beating.

## Materials and methods

### Preparation of OAD (22S dynein)

OAD was purified from *T. thermophila* (strain B-255) cilia as previously described^18^. Cells were grown in 1% (w/v) proteose peptone No. 3 (Thermo Fisher Scientific, Waltham, MA), 0.5% (w/v) yeast extract (Thermo Fisher Scientific, Waltham, MA), 0.87% (w/v) glucose (Wako Pure Chemical, Osaka, Japan), 0.001% Ampicillin (Wako Pure Chemical, Osaka, Japan), and 0.03% antifoaming agent (FS Antifoam DB-110N, Dow Corning Toray, Tokyo, Japan) in 5 L Fernbach flasks with aeration at 37 °C. Cilia were isolated using the Ca^2+^ shock method^33^. The cilia were demembranated using 0.3% Nonidet-P40 (Sigma-Aldrich, St. Louis, MO) in Prep buffer (10 mM HEPES, 4 mM MgSO_4_, 0.1 mM EGTA, 100 mM NaCl, pH 7.4) supplemented with protease inhibitors for 30 min on ice, and centrifuged at 12,000*g* for 15 min at 4 °C. The isolated axonemes were resuspended in Prep buffer supplemented with 0.6 M NaCl and 1 mM phenylmethylsulfonyl fluoride (PMSF), rested for 30 min on ice, and centrifuged at 40,000*g* for 10 min at 4 °C. The supernatant was fractionated in 30-mL of a 5–20% (w/w) sucrose density gradient in Prep buffer containing 0.1 mM ATP (Grade II, Sigma-Aldrich, St. Louis, MO) and 0.1 mM PMSF. After sedimentation at 89,000*g* for 16 h at 2 °C in a centrifuge with a P28S ultracentrifuge rotor (CP70MX, Hitachi Koki, Tokyo, Japan), the gradient was separated into 20 fractions, and the fractions containing the OAD protein peak were pooled. The pooled samples were further purified using an anion-exchange column (Mono Q 5/50 GL, Cytiva, Marlborough, MA), and concentrated using an ultrafiltration unit (Amicon Ultra-100 K, Millipore, Billerica, MA). Purified OAD was aliquoted, flash-frozen, and stored in liquid nitrogen. The concentration of OAD was estimated by SDS-PAGE on 7.5% acrylamide gels using bovine serum albumin (BSA) standards (Thermo Fisher Scientific, Waltham, MA) loaded on the same gel. The gels were stained with Quick-CBB PLUS (Wako Pure Chemical, Osaka, Japan) and imaged using a CCD camera (CSFX36BC3, Toshiba-teli, Tokyo, Japan). The bands containing OAD and BSA standards were quantified using the Quantity One software (Bio-Rad, Hercules, CA).

### Purification of tubulin and polymerization of microtubules

Tubulin was purified from porcine brain by three cycles of depolymerization and polymerization followed by phosphocellulose chromatography^34^. Purified tubulin was aliquoted and flash-frozen and stored in liquid nitrogen. For the microbead motility assay, biotinylated (biotin-(AC_5_)_2_-Sulfo-OSu, Dojindo, Kumamoto, Japan) and Alexa 488-labeled (Alexa Fluor 488 succinimidyl ester, Thermo Fisher Scientific, Waltham, MA) microtubules were prepared by polymerization of a mixture of biotinylated, Alexa488-labeled, and non-fluorescent tubulin in a molar ratio of 15 : 1 : 30–50 in BRB80 buffer (80 mM PIPES, 1 mM MgCl_2_, 1 mM EGTA, pH 6.8) supplemented with 1 mM GTP and 1 mM MgCl_2_ for 30 min at 37 °C, and then stabilized by adding 40 µM taxol (Sigma-Aldrich, St. Louis, MO)^28^. For the in vitro microtubule gliding assay, rhodamine-labeled (X-rhodamine succinimidyl ester, Thermo Fisher Scientific, Waltham, MA) microtubules were prepared by polymerization of a mixture of rhodamine-labeled and non-fluorescent tubulin in a molar ratio of 1 : 16 in BRB80 with 1 mM MgCl_2_ and 1 mM GTP for 30 min at 37 °C and then stabilized by adding 20 µM taxol^35^.

### Preparation of dynein-coated beads

OAD coated 0.1-µm diameter carboxylate-modified polystyrene microbeads (FluoSphere, red fluorescent (580/605), Thermo Fisher Scientific, Waltham, MA) were prepared. First, 5 µL of 0.6-nM microbeads were incubated with 5 µL of 170 nM OAD solution in 50 K-Ace buffer (10 mM PIPES, 50 mM potassium acetate, 4 mM MgSO_4_, 1 mM EGTA, pH 7.4) for 2 min on ice, and then 0.5 µL of 11 mg mL^−1^ casein (Nacalai Tesque, Kyoto, Japan) was added to prevent further non-specific adsorption. After collection by centrifugation, the microbeads were re-suspended into the 50 K-Ace buffer (100 µL) containing 0.5 mg mL^−1^ casein, 10 µM ATP, 20 µM taxol, ATP regeneration (creatine kinase (Roche, Basel, Switzerland), creatine phosphate (Roche, Basel, Switzerland)) and oxygen scavenger systems (catalase (Roche, Basel, Switzerland), glucose oxidase (Sigma-Aldrich, St. Louis, MO) and glucose). The re-suspended microbeads were observed for approximately 30 min. In a preliminary screening for a condition that allows to observe stable motion of the microbeads along the microtubule, excessive amounts of OAD molecules required mixing with the microbeads in a molar ratio of 1 : 280. We estimated the maximum number of OAD molecules attached to each microbead to be approximately ten (half the surface area of a microbead / occupied area of OAD ≈ 1.6 × 10^4^ nm^2^/30 × 50 nm^2^), although we could not exactly determine the number of simultaneously interacting OAD molecules with a microtubule at any given time.

### 3D-motility of ODA-coated microbeads around a suspended microtubule

A 3D prismatic optical tracking (*tPOT*) microscope was constructed as previously described^18,25,28^. The flow chamber was prepared according to the method described by Maruyama et al.^28^. The chamber was assembled with 20 × 30 mm patterned glass coverslips (150 μm thick; Matsunami Glass. Ind., Osaka, Japan) and 18 × 18 mm unpatterned glass coverslips (130–170 μm thick; Matsunami Glass, Ind., Osaka, Japan) using two thin strips of tape (810-1-18, Scotch 3 M) placed with a 5 mm gap as spacers inside two parallel lines of grease (Apiezon M Grease, M&I Materials, Manchester, UK). Microgrooves on the patterned glass coverslip were fabricated using a standard photolithography technique^28,36^. Parallel walls on the patterned glass allowed microtubules to suspend away from the glass surface. First, the flow chamber was incubated with two chamber volumes of 5 mg mL^−1^ biotinylated-BSA (Sigma-Aldrich, St. Louis, MO) in 50 K-Ace buffer for 3 min, then rinsed with two chamber volumes of cold 50 K-Ace buffer, and incubated for 3 min with two chamber volumes of 2.5 mg mL^−1^ streptavidin (Sigma-Aldrich, St. Louis, MO) in 50 K-Ace buffer at room temperature. Following a rinse with two chamber volumes of W buffer (50 K-Ace buffer supplemented with 0.5 mg mL^−1^ casein and 10 µM taxol), two chamber volumes of 0.1 mg mL^−1^ Alexa488-biotin-labeled microtubules were loaded and incubated for 3 min at room temperature. The non-immobilized microtubules were washed away with two chamber volumes of W buffer. Finally, the chamber was loaded with two chamber volumes of OAD-coated microbead solution containing 10 μM ATP (BioXtra, Sigma-Aldrich, St. Louis, MO), ATP regeneration and oxygen scavenger systems, 1 mM dithiothreitol (DTT, Roche, Basel, Switzerland), and 20 μM taxol, sealed with grease, and mounted on the *tPOT* microscope to measure the microbead motion at room temperature (24 ± 1 °C). Fluorescence was passed through an appropriate filter set (for Alex488-labeled microtubules, GFP filter set, Semrock, Rochester, NY, and for fluorescent red microbead, G2A set, Nikon, Tokyo, Japan). Microbeads on a straight microtubule were tracked every 0.1 s with the *tPOT* system to track their 3D positions (*x*: parallel to the longitudinal axis, *z*: perpendicular to the imaging plane, *y*: perpendicular to both *x* and *z*; Fig. 1a). The position of the two optically separated images of a microbead from an identical microbead were determined by 2D Gaussian fitting as (*x*_1_, *y*_1_) and (*x*_2_, *y*_2_), and *x*, y, and *z* was calculated by (*x*_1_ + *x*_2_)/2, (*y*_1_ + *y*_2_)/2 and *y*_1_−*y*_2_, respectively. For calibration of the real *z*-axis position and *y*_1_−*y*_2_, a custom-built stable stage (Chukousya Seisakujo, Tokyo, Japan) equipped with a pulse motor (SGSP-13ACTR-BO, Sigma Koki, Tokyo, Japan) and controller (QT-CM2, Chuo Precision Industrial, Tokyo, Japan) was used to move the objective vertically while observing the stable microbead (0.1 µm in diameter, Thermo Fisher Scientific) bound to the suspended microtubule. The calculated *z-*position and actual *z-*position (as defined by the pulse motor) corresponded linearly over a range of ± 0.5 µm from the focal plane. We analyzed microbeads that exhibited stable motion (> 0.9 µm) along a stably suspended single microtubule. Those on fluctuating, bundled, or crossed microtubules, and those on a non-suspended part of a microtubule, that is, the segment bound on the patterned hilltop, were ignored. Microbeads that encountered other microbeads and those that exhibited a helical trajectory whose radius was too large for some unknown reason (> 250 nm, ~ twice as large as the sum of the radii of the microtubule and the microbead) were also excluded. Finally, we obtained 32 trajectories. All these showed helical motion around the microtubules. The observed pitch (*P*_obs_) comprised two helical elements: the helical motion driven by dyneins (*P*_mot_) around the microtubule surface, and the supertwist of the microtubule depending on the number of the protofilaments (*P*_MT_, 7–8 µm for the 14-protofilament microtubule, but none for the 13-protofilament microtubule). Based on the well-known relationship between them, *P*_mot_^−1^ = *P*_obs_^−1^ − *P*_MT_^−1^^21,22,28,37^. According to our previous study, microtubule polymerization with 1 mM GTP in BRB80 buffer formed a 5 : 3 mixture of 13- and 14-protofilament microtubules^25^. The difference between *P*_obs_ and *P*_mot_ in proportion of the 13-protofilament microtubules in the mixture to the 14-protofilament microtubules of our preparation was less than 10%. Thus, the values of the observed pitch without correction were used in this study. *xyz* trajectories were determined using measurements from at least three independent assays.

### Microtubule gliding assay

Microtubule gliding assays were performed in flow chambers assembled from two coverslips attached together using double-sided tape (NW-25, Nichiban, Tokyo, Japan). The chamber was first incubated with one chamber volume of 0.03 μM dynein solution for 3 min. The chamber was filled with four chamber volumes of 1 mg mL^−1^ casein in 50 K-Ace buffer for 2 min to coat the glass surface and then rinsed with four chamber volumes of 50 K-Ace buffer. Two volumes of rhodamine-labeled microtubules (~ 1 μg mL^−1^) in BRB80 buffer supplemented with 20 μM taxol and 0.4 mg mL^−1^ casein were added and incubated for 2 min, and then rinsed with two chamber volumes of 50 K-Ace buffer. Finally, two chamber volumes of 50 K-Ace buffer containing 10 μM ATP, ATP regeneration and oxygen scavenger systems, 1 mM DTT, and 20 μM taxol were introduced to the chamber. The chamber was sealed with a nail varnish. Assays were performed at room temperature (24 ± 1 °C). Microtubule gliding was observed using an inverted fluorescence microscope (TE2000-U, Nikon, Tokyo, Japan) with a custom-built stable stage (KS-N) and a stage controller (QT-CM2-35), using illumination from a mercury lump (Intensilight, Nikon, Tokyo, Japan), 100× /1.49 NA, Plan-Apochromat objective lenses (Nikon, Tokyo, Japan), and G2A filter set. A reduction lens (0.6×) was inserted into the camera port on the right to widen the field of view. Images of microtubule gliding driven by OAD molecules coated with the lower cover glass composing the flow chamber were recorded using an EMCCD camera (iXon X_3_ DU-897E-COO-#BV, Andor Technology, Belfast, UK) via the Solis software (Andor Technology, Belfast, UK) with a 5-s exposure for a 60-s imaging cycle. The positions of the leading tip of the microtubule were obtained manually using tracking software (Mark2, kindly provided by Dr. Ken’ya Furuta, NICT)^38^. The positions of the tip of the gliding microtubules were determined using measurements from at least three independent assays.

## Supplementary Information

Supplementary Video 1.

Supplementary Video 2.

Supplementary Figures.

Supplementary Legends.

## Data Availability

All samples used in this study are available from the corresponding authors on reasonable request. The source data for graphs in the figures during the current study are provided as Supplementary Data 1.

## References

[1] Satir P (1968). Studies on cilia. 3. Further studies on the cilium tip and a ‘sliding filament’ model of ciliary motility. J. Cell Biol..

[2] Gibbons IR (1963). Studies on the protein components of cilia from *Tetrahymena pyriformis*. Proc. Natl. Acad. Sci. USA.

[3] Seetharam RN, Satir P (2008). Coordination of outer arm dynein activity along axonemal doublet microtubules. Cell Motil. Cytoskelet..

[4] Johnson KA, Wall JS (1983). Structure and molecular weight of the dynein ATPase. J. Cell Biol..

[5] Tang WJ, Bell CW, Sale WS, Gibbons IR (1982). Structure of the dynein-1 outer arm in sea urchin sperm flagella. I. Analysis by separation of subunits. J. Biol. Chem..

[6] Pigino G (2012). Comparative structural analysis of eukaryotic flagella and cilia from Chlamydomonas, Tetrahymena, and sea urchins. J. Struct. Biol..

[7] Nicastro D (2006). The molecular architecture of axonemes revealed by cryoelectron tomography. Science.

[8] Walton T, Wu H, Brown A (2021). Structure of a microtubule-bound axonemal dynein. Nat. Commun..

[9] Carter AP (2008). Structure and functional role of dynein’s microtubule-binding domain. Science.

[10] King SM, Sale WS (2018). Fifty years of microtubule sliding in cilia. Mol. Biol. Cell.

[11] Goodenough U, Heuser J (1984). Structural comparison of purified dynein proteins with in situ dynein arms. J. Mol. Biol..

[12] Toyoshima YY (1987). Chymotryptic digestion of Tetrahymena 22S dynein. I. Decomposition of three-headed 22S dynein to one- and two-headed particles. J. Cell Biol..

[13] Kubo S (2021). Remodeling and activation mechanisms of outer arm dyneins revealed by cryo-EM. EMBO Rep..

[14] Rao Q (2021). Structures of outer-arm dynein array on microtubule doublet reveal a motor coordination mechanism. Nat. Struct. Mol. Biol..

[15] Vale RD, Toyoshima YY (1988). Rotation and translocation of microtubules in vitro induced by dyneins from *Tetrahymena cilia*. Cell.

[16] Furuta A, Yagi T, Yanagisawa H-A, Higuchi H, Kamiya R (2009). Systematic comparison of in vitro motile properties between Chlamydomonas wild-type and mutant outer arm dyneins each lacking one of the three heavy chains. J. Biol. Chem..

[17] Hirakawa E, Higuchi H, Toyoshima YY (2000). Processive movement of single 22S dynein molecules occurs only at low ATP concentrations. Proc. Natl. Acad. Sci. USA.

[18] Yamaguchi S (2015). Torque generation by axonemal outer-arm dynein. Biophys. J..

[19] Kagami O, Kamiya R (1992). Translocation and rotation of microtubules caused by multiple species of Chlamydomonas inner-arm dynein. J. Cell Sci..

[20] Vale RD (2003). The molecular motor toolbox for intracellular transport. Cell.

[21] Mitra A, Ruhnow F, Nitzsche B, Diez S (2015). Impact-free measurement of microtubule rotations on kinesin and cytoplasmic-dynein coated surfaces. PLoS ONE.

[22] Can S, Dewitt MA, Yildiz A (2014). Bidirectional helical motility of cytoplasmic dynein around microtubules. Elife.

[23] Kikushima K, Kamiya R (2008). Clockwise translocation of microtubules by flagellar inner-arm dyneins in vitro. Biophys. J..

[24] Fujimura S, Ito Y, Ikeguchi M, Adachi K, Yajima J (2017). Biochemical and biophysical research communications dissection of the angle of single fluorophore attached to the nucleotide in corkscrewing microtubules. Biochem. Biophys. Res. Commun..

[25] Yajima J, Mizutani K, Nishizaka T (2008). A torque component present in mitotic kinesin Eg5 revealed by three-dimensional tracking. Nat. Struct. Mol. Biol..

[26] Katoh TA (2018). Three-dimensional tracking of microbeads attached to the tip of single isolated tracheal cilia beating under external load. Sci. Rep..

[27] Marumo A, Yamagishi M, Yajima J (2021). Three-dimensional tracking of the ciliate Tetrahymena reveals the mechanism of ciliary stroke-driven helical swimming. Commun. Biol..

[28] Maruyama Y (2021). CYK4 relaxes the bias in the off-axis motion by MKLP1 kinesin-6. Commun. Biol..

[29] Shiroguchi K, Toyoshima YY (2001). Regulation of monomeric dynein activity by ATP and ADP concentrations. Cell Motil. Cytoskelet..

[30] Ferro LS, Can S, Turner MA, Elshenawy MM, Yildiz A (2019). Kinesin and dynein use distinct mechanisms to bypass obstacles. Elife.

[31] Mitra A, Ruhnow F, Girardo S, Diez S (2018). Directionally biased sidestepping of Kip3/kinesin-8 is regulated by ATP waiting time and motor–microtubule interaction strength. Proc. Natl. Acad. Sci..

[32] Reck-Peterson SL (2006). Single-molecule analysis of dynein processivity and stepping behavior. Cell.

[33] Rosenbaum JL, Carlson K (1969). Cilia regeneration in Tetrahymena and its inhibition by colchicine. J. Cell Biol..

[34] Weingarten MD, Suter MM, Littman DR, Kirschner MW (1974). Properties of the depolymerization products of microtubules from mammalian brain. Biochemistry.

[35] Yajima J, Alonso MC, Cross RA, Toyoshima YY (2002). Direct long-term observation of kinesin processivity at low load. Curr. Biol..

[36] Ahamed MJ, Senkal D, Trusov AA, Shkel AM (2015). Study of high aspect ratio NLD plasma etching and postprocessing of fused silica and borosilicate glass. J. Microelectromech. Syst..

[37] Brunnbauer M (2012). Torque generation of kinesin motors is governed by the stability of the neck domain. Mol. Cell.

[38] Furuta K, Toyoshima YY (2008). Minus-end-directed motor ncd exhibits processive movement that is enhanced by microtubule bundling in vitro. Curr. Biol..

