# Supplementary material for: Torque generating properties of Tetrahymena ciliary three-headed outer-arm dynein
Source: Sci Rep. 2022 Oct 6;12:16722. doi: 10.1038/s41598-022-21001-0 (PMC9537190; doi:10.1038/s41598-022-21001-0)
Supplement: Supplementary file 4 — Supplementary Legends. [file 41598_2022_21001_MOESM4_ESM.pdf]

Supplementary Information for

Torque generating properties of *Tetrahymena* ciliary three-headed outer-arm dynein

Shin Yamaguchi [a, #], Masahiko Yamagishi [a, #], Junichiro Yajima [a,b,c,d, ‡]

[a] *Department of Life Sciences, Graduate School of Arts and Sciences, The University of Tokyo, 3-8-1 Komaba, Meguro-ku, Tokyo 153-8902, Japan.*

[b] *Komaba Institute for Science, The University of Tokyo, 3-8-1 Komaba, Meguro-ku, Tokyo 153-8902, Japan*

[c] *Research Center for Complex Systems Biology, The University of Tokyo, 3-8-1 Komaba, Meguro-ku, Tokyo 153-8902, Japan.*

[d] *Universal Biological Institute, The University of Tokyo, Bunkyo-ku, Tokyo 113-0033, Japan*

[#] indicates equal contribution.

[‡] Correspondence should be addressed to J.Y. (yajima@bio.c.u-tokyo.ac.jp)

Supplementary movies:

Supplementary Movie 1 OAD-coated microbead translocated along the suspended microtubule. The two fields divided by the white dotted line are a pair of split images by the prism. The orange open square indicates a suspended microtubule across a 10-µm gap between the wall (gray lines). The solid and open red arrowhead indicate the image of the microbead split by the prism. This movie is 15 seconds long at normal speed. This image shows a region 40 × 4 µm in size. Scale bar: 2 µm.

Supplementary Movie 2 Circumferential gliding movement of the microtubule driven by OAD. The gliding microtubule driven by OAD molecules fixed to cover glass surface tends to turn to the left, resulting in a curved path. This movie is 5 seconds long at ×60 speed. This image shows a region 47 × 47 µm in size. Scale bar: 10 µm.
